# The invisibility of the cognitive cost of military police work

**DOI:** 10.1590/1980-220X-REEUSP-2023-0329en

**Published:** 2024-05-20

**Authors:** Rejane de Fátima Parada Viegas, Karla Gualberto Silva, Adriana de Oliveira Sarefino, Eloá Carneiro Carvalho, Angela Maria Mendes Abreu, Pedro Miguel Santos Dinis Parreira, Norma Valéria Dantas de Oliveira Souza, Regina Célia Gollner Zeitoune, Sheila Nascimento Pereira de Farias

**Affiliations:** 1 Universidade Federal do Rio de Janeiro, Escola de Enfermagem Anna Nery, Programa de Pós-Graduação Stricto Sensu em Enfermagem, Rio de Janeiro, RJ, Brazil.; 2 Escola Nacional de Saúde Pública Sergio Arouca, Programa de Pós-Graduação em Bioética, Ética Aplicada e Saúde Coletiva, Rio de Janeiro, RJ, Brazil.; 3 Escola Superior de Enfermagem de Coimbra, Coimbra, Portugal.; 4 Universidade do Estado do Rio de Janeiro, Faculdade de Enfermagem, Programa de Pós-Graduação Stricto Sensu em Enfermagem, Rio de Janeiro, RJ, Brazil.

**Keywords:** Occupational Health, Police, Nursing, Salud Ocupacional, Policia, Enfermería, Saúde Ocupacional, Polícia, Enfermagem

## Abstract

**Objective:**

To assess the cognitive cost of work for military police officers in the state of Rio de Janeiro.

**Method:**

This is a cross-sectional study with a quantitative approach, carried out with 446 military police officers, of both sexes, distributed between non-commissioned officers and officers, in the 7th, 15th, 20th, 24th and 41st Military Police Battalions. An instrument was used to depict sociodemographic, work, lifestyle and health conditions and a scale for assessing the human cost of work, which analyses the demands of the job through physical, cognitive and affective costs. The data was organized, processed and analyzed using the Statistical Package for the Social Sciences (SPSS) software, version 13.1.

**Results:**

The cognitive cost had the highest means, with severe results (μ = 3.86; SD = 0.86), representing greater demands in relation to the human cost of work among military police officers in the state of Rio de Janeiro and significant associations in relation to obesity, cognitive alterations in attention and memory, age and hours of sleep.

**Conclusion:**

In assessing the human cost of work, the cognitive cost was the most demanding in the work context of the military police officers surveyed, presenting a serious risk of illness.

## RESUMO

**Objetivo:**

Analisar o custo cognitivo no trabalho dos policiais militares do estado do Rio de Janeiro.

**Método:**

Trata-se de um estudo de corte transversal com abordagem quantitativa, realizado com 446 policiais militares, de ambos os sexos, distribuídos entre praças e oficiais, nos 7°, 15°, 20°, 24° e 41° batalhões de Polícia Militar. Utilizou-se um instrumento para a caracterização sociodemográfica, laboral, hábitos de vida e condições de saúde e uma escala de avaliação do custo humano no trabalho, que analisa as exigências relativas ao trabalho por meio dos custos físico, cognitivo e afetivo. Os dados foram organizados, processados e analisados com o auxílio do programa *Statistical Package for the Social Sciences* (SPSS), versão 13.1.

**Resultados:**

O custo cognitivo apresentou as maiores médias, com resultados graves (μ = 3,86; DP = 0,86), representando maior exigência em relação ao custo humano no trabalho entre os policiais militares do estado do Rio de Janeiro e associações significativas em relação à obesidade, alterações cognitivas de atenção e memória, idade e horas de sono.

**Conclusão:**

Na avaliação do custo humano no trabalho, o custo cognitivo foi o mais exigido no contexto de trabalho dos policiais militares pesquisados apresentando um risco grave para o adoecimento.

## RESUMEN

**Objetivo:**

Analizar los costos cognitivos producidos por el trabajo de los policías militares del estado de Rio de Janeiro.

**Método:**

Se trata de un estudio transversal con abordaje cuantitativo, realizado con 446 policías militares, de ambos sexos, distribuidos entre cuadros y oficiales, de los Batallones 7°, 15°, 20°, 24° y 41° de la Policía Militar. Se utilizó un instrumento para caracterizar las condiciones sociodemográficas, laborales, de estilo de vida y de salud, y una escala para evaluar el costo humano del trabajo, que analiza las exigencias del trabajo a través de los costos físicos, cognitivos y afectivos. Los datos fueron organizados, procesados y analizados utilizando el software Statistical Package for the Social Sciences (SPSS), versión 13.1.

**Resultados:**

El costo cognitivo presentó las medias más altas, con resultados graves (μ = 3,86; SD = 0,86), representando mayores exigencias en relación al costo humano del trabajo entre los policías militares del estado de Río de Janeiro y asociaciones significativas en relación a la obesidad, alteraciones cognitivas de atención y memoria, edad y horas de sueño.

**Conclusión:**

Al evaluar el coste humano del trabajo, el coste cognitivo fue el más exigente en el contexto laboral de los policías militares encuestados, presentando un grave riesgo de enfermedad.

## INTRODUCTION

The analysis of working conditions in the contemporary world should not overlook the fact that globalization of pecuniary order and the precariousness of the social environment, in dialogue with innovations in technology and management, have impacted multiple transformations in the traditional ideology of the areas of Occupational Medicine and Occupational Health^(1)^.

Furthermore, with regard to the aspect of work as a mediator of social integration, whether through the constitution of subjectivity from an economic or cultural perspective, it is accepted that priority should not only be given to the physical aspects of work environments as risk factors for workers’ health, but also to organizational factors and, above all, the process of psychosocial formation of workers in their professional fullness.

Against this backdrop, depending on the type of organization and work process to which the worker is inserted, the activities carried out can exceed their physical limits, especially when exposed to biomechanical and biological risks and risks to mental health, a scenario that characterizes functional wear and tear. Thus, the way in which the work process is organized can lead to a deterioration in mental health^(2)^.

The demands required to workers have been studied by various authors over the years^(3–5)^. However, the lack of clarity in the roles assigned and the demands for new skills imply a human cost that needs to be assessed, since they have an impact on workers’ health.

There is a gap between what is imposed on the worker in the job description (prescribed work) and the activity that arises from unforeseen events (real work). In this sense, both public and private organizations should focus on human resources management policies and employee development and behavior. These activities are important for developing skills that allow for better integration of different areas of knowledge, achieving results for the organization without overloading teams^(6)^.

Faced with the conflict between what is real and what is prescribed, work organization needs to provide autonomy and freedom so that workers can face unusual situations creatively and thus strengthen their identity. On the other hand, the unexpected generates suffering and the way in which this worker manages to deal with unexpected situations will depend on the configuration of the work organization^(7)^.

For these reasons, when it is characterized as rigid in its norms and rules, and does not give workers a certain amount of autonomy and freedom to seek solutions to problems, it results in an increase in the state of suffering. It should be noted that suffering is inevitable in the context of life and, in turn, at work, but when it becomes intense and repetitive, it can generate pathogenic suffering, which has the potential to make workers ill^(8)^.

On the other hand, rigidity that doesn’t give workers autonomy results in human costs, such as physical, cognitive and emotional strain, imposed when carrying out work activities. It should be noted that the cognitive cost is the intellectual effort required to learn, as well as the ability to solve problems and make decisions at work^(9)^. However, among the professions with high cognitive demands that result in illness, there is the need to highlight the situation of teachers, journalists, doctors, nurses and police officers^(10)^.

In this context, and given the problems surrounding cognitive demands in the context of police work, as well as their importance in an individual and collective sense, this study was directed towards the analysis of the cognitive cost of work for military police officers in the state of Rio de Janeiro.

## METHOD

### Type of Study

This is a cross-sectional study with a quantitative approach, based on the Strengthening the Reporting of Observational Studies in Epidemiology (STROBE) guidelines for writing the manuscript.

Considering the high crime rates presented at the National Symposium on Police Victimization in the State of Rio de Janeiro, held by the Military Police (PM in the Portuguese acronym) in 2019^(11)^, the battalions with the most armed confrontations in Metropolitan Region I of Rio de Janeiro were chosen as the study sites for this thesis.

The sample, by battalion, was made up of 446 military police officers of both sexes, distributed among the ranks (privates, corporals, sergeants and deputy lieutenants) and patents (lieutenant, captain, major, lieutenant colonel and colonel), members of a population of police officers in the 7th, 15th, 20th, 24th and 41st Military Police battalions (BPM in the Portuguese acronym) in the state of Rio de Janeiro.

The inclusion criterion was all military personnel assigned to the chosen battalions who were physically and mentally able to answer the questionnaire at the time of the interview. The exclusion criteria were inactive military personnel, those on vacation and those undergoing health treatment without rehabilitation.

### Sample Calculation

The sample size was calculated from the population of 44,336 police officers. To determine the sample size, the EPI-INFO program version 7.2.2.16 was used, establishing an error of 5.0%, reliability of 95.0%, an expected proportion of 50.0% and a loss percentage of 20.0%.

After calculating the sample stratified by battalion (n = 417), the study participants were invited and there were no refusals when approached. However, there were other police officers who, during data collection, also wanted to take part in the study and were incorporated into the sample, considering that they met the inclusion criteria, totaling n = 446 participants, distributed as follows by battalion: 7th BPM n = 108; 15th BPM n = 104; 20th BPM n = 98; 24th BPM n = 54; 41 BPM n = 82.

### Data Collection

Data was collected by spontaneous demand/convenience, between December 2021 and June 2022, using two instruments. The instruments were filled in by the police officer himself, and the deadline for returning the material was set for the following shift. In cases where the material was not returned within the specified timeframe, a new contact was made with the participant and a new date was set for the return of the data collection instruments, with no loss of participants.

The first instrument consisted of self-reported questions to characterize sociodemographics, health and work. Among the sociodemographic, work and health variables selected for this study were gender, age, rank, battalion, length of service in the Military Police, comorbidities, reasons for sick leave and hours of sleep. No pilot test was carried out.

The second instrument was a scale from the Inventory of Work and Illness Risks (ITRA in the Portuguese acronym). The Inventory of Work and Risk of Illness is a self-administered questionnaire that assesses the dimensions of work that pose a risk of illness, pointing out critical indicators that are related to work and is made up of four scales: the Work Context Assessment Scale (EACT in the Portuguese acronym), the Human Cost of Work Assessment Scale (EACHT in the Portuguese acronym), the Indicators of Pleasure and Suffering at Work Scale (EIPST in the Portuguese acronym) and the Work-Related Injury Assessment Scale (EADRT in the Portuguese acronym), and their effects on workers’ health^(12)^.

The instrument was validated in Brazil in 2003 by Ferreira and Mendes, adapted and revalidated in 2006 and published by Mendes in 2007. It was created to meet the demand for research into the relationship between work and the risk of illness^(12)^.

In order to meet the objective of the study, the Human Cost at Work Assessment Scale, (EACHT) was used, which analyzes the demands of work through the following costs: Physical cost (questions 1 to 10); Cognitive cost (questions 11 to 20); Affective cost (questions 21 to 30), made up of five points, in which: “1 = not at all required, 2 = not very much required, 3 = more or less required, 4 = very much required, 5 = totally required”, following the same risk classification recommendations. The scale has an eigenvalue greater than 2, a total variance of 44.98%, a KMO of 0.91 and 50% of the correlations above 0.30^(12)^.

The results of the EACHT were interpreted following the guidelines of Mendes (2007)^(11)^ and criteria were used to classify the risk of the factors in the scale: when the averages were: above 3.7 – the most negative evaluation, serious risk of illness; between 3.69 and 2.3 – moderate evaluation, critical risk of illness; and below 2.29 – positive/satisfactory evaluation, the work environment favors the health of the professional.

### Data Analysis

In order to organize and tabulate the database, the study used SPSS (Statistical Package for the Social Sciences) for Windows and Excel 365 software, with double typing to minimize typing errors and inconsistencies. All the tests were applied with a 95% confidence level and calculated taking into account valid answers, i.e. ignored answers were not taken into account.

The results are presented in table form with their respective absolute and relative frequencies. The numerical variables are represented by the measures of central tendency and measures of dispersion of the sociodemographic and work characteristics and the items on the Human Cost of Work Assessment Scale.

The Chi-Square Test and Fisher’s Exact Test were used to assess statistically significant associations. To test for normality, the Kolmogorov-Smirnov non-parametric test was used for the quantitative variables.

In order to test the comparison of three or more independent samples, the Kruskal-Wallis non-parametric test was used.

### Ethical Aspects

The research was submitted to the Ethics and Research Committee of the Anna Nery School of Nursing (CEP da EEAN), São Francisco de Assis Primary Care Institute, of the Federal University of Rio de Janeiro, UFRJ, in accordance with Resolution 466/2012 and cleared by the substantiated opinion number: 5.137.132.

The participants were also given the Informed Consent Form (ICF) along with the questionnaires. At this point, the objectives of the research were presented and the participants were read the Informed Consent Form in order to clarify any doubts; finally, they were asked to sign it. Participation in the study was voluntary.

## RESULTS

A total of 446 participants took part in the study, the majority of whom were men (86.7%, n = 379), with an average age of 37 (38.5%), married (78%, n = 311) and with one child (4%, n = 137). There was a higher rate of participation in this study among soldiers (privates, corporals, sergeants and sub-lieutenants) (94%, n = 420) compared to officers (4.5%, n = 20), with an average of 10 years’ service.

According to the characterization of these workers, an average of six (6) hours of sleep per day was observed (24.7%), and 29.7% (n = 110) reported cognitive impairment related to memory.

In the assessment of the Human Cost of Work, among the factors, the most negative evaluations were for cognitive cost, with 65% (n = 282) having a severe negative evaluation, indicating that the work context poses risks of illness, as shown in Table 1.

**Table 1:** Distribution of risk classification in the Human Cost of Work Assessment Scale (n = 446) – Rio de Janeiro, RJ, Brazil, 2023.

Variables	Severen (%)	Criticaln (%)	Satisfactoryn (%)
**EACHT**			
Physical cost	86 (19,8)	**257 (59,2)**	91 (21,0)
Cognitive cost	**282 (65,0)**	126 (29,0)	26 (6,0)
Affective cost	149 (34,3)	**190 (43,8)**	95 (21,9)

*There were participants who did not answer some of the factor items.

As can be seen in Table 2, among the factors of the Human Cost of Work Assessment Scale, cognitive cost had the highest average, with serious results (μ = 3.86; SD = 0.86), representing greater demands in the work context of this population.

**Table 2:** Distribution of responses according to the factors of the Human Cost at Work according to the presence of illness at work (n = 446) – Rio de Janeiro, RJ, Brazil, 2023.

Variables	Mean (μ)	SD	Median	Percentile 25	Percentile 75	Minimum	Maximum
Physical Cost	2,91	0,85	2,85	2,30	3,40	1,10	5,00
Cognitive Cost	3,86	0,86	4,00	3,30	4,60	1,00	5,00
Affective Cost	3,15	1,06	3,10	2,40	4,00	1,00	5,00

Table 3 shows that the most negative items in the Cognitive Cost were: “having to solve problems” (μ = 4.09; SD = 1.02); “being forced to deal with unforeseen events” (μ = 4.20; SD = 0.98); “using vision continuously” (μ = 3.85; SD = 1.18); “using memory” (μ = 4.12; SD = 1.01); “having intellectual challenges” (μ = 3.78; SD = 1.14); “making a mental effort” (μ = 3.93; SD = 1.12); “having mental concentration” (μ = 4.15; SD = 0.98); “using creativity” (μ = 3.94; SD = 1.07).

**Table 3:** Distribution of the mean, standard deviation and risk classification for each item that makes up the Cognitive Cost factor (n = 446) – Rio de Janeiro, RJ, Brazil, 2023.

Human cost at work	Mean ± SD	Risk classification
Cognitive Cost		
Developing tricks	2,86 ± 1,43	Critical
Having to solve problems	4,09 ± 1,02	Severe
Having to deal with unforeseen events	4,20 ± 0,98	Severe
Predicting events	3,68 ± 1,21	Critical
Continuous use of vision	3,85 ± 1,18	Severe
Use your memory	4,12 ± 1,01	Severe
Have intellectual challenges	3,78 ± 1,14	Severe
Making a mental effort	3,93 ± 1,12	Severe
Mental concentration	4,15 ± 0,98	Severe
Use creativity	3,94 ± 1,07	Severe

With regard to the association of cognitive cost with sociodemographic variables, work, lifestyle habits and health conditions and cognitive alterations (Table 4), there was a statistically significant association between the variables “Obesity”, “Attention” and “Memory” with severe risk classification. In the numerical variables, there was a statistically significant difference in “Age” and “Hours of sleep per day”. Although the “Anxiety Crisis” variable had a p-value greater than 0.05, it was very close to being significant and, when taking into account the objectives of the study, it is important to highlight this result as well.

**Table 4:** Association between cognitive cost and sociodemographic variables (personal, work and health conditions) (n = 446) – Rio de Janeiro, RJ, Brazil, 2023.

Variables**	EACHT – cognitive cost			P-value
	Severe n (%)	Critical n (%)	Satisfactory n (%)	
**Sex**				
Women	37 (64,9)	17 (29,8)	3 (5,3)	0,957*
Men	238 (64,7)	107 (29,1)	23 (6,3)	
**Rank**				
Private	267 (65,4)	115 (28,2)	26 (6,4)	0,453*
Officer	13 (65,0)	7 (35,0)	0 (0,0)	
**Military organization**				
7 BPM	56 (58,3)	31 (32,3)	9 (9,4)	0,343*
15 BPM	60 (64,5)	28 (30,1)	5 (5,4)	
20 BPM	63 (70,0)	22 (24,4)	5 (5,6)	
24 BPM	26 (54,1)	19 (39,6)	3 (6,3)	
41 BPM	56 (71,8)	20 (25,6)	2 (2,6)	
**Comorbidities**				
High blood pressure	42 (67,7)	16 (25,8)	4 (6,5)	0,831*
Cardiopathy	5 (62,5)	3 (37,5)	0 (0,0)	0,825**
Obesity	40 (88,9)	5 (11,1)	0 (0,0)	0,002*
**Have you ever considered suicide**				
Yes	47 (72,3)	16 (24,6)	2 (3,1)	0,350*
No	232 (63,9)	108 (29,8)	23 (6,3)	
**Reason for leave permit**				
Depression	48 (75,0)	12 (18,7)	4 (6,3)	0,124*
Anxiety crisis	62 (76,5)	16 (19,8)	3 (3,7)	0,053*
Bipolar disorder	14 (87,5)	2 (12,5)	0 (0,0)	0,209**
Low back pain	22 (78,6)	4 (14,3)	2 (7,1)	0,206*
Myalgia (muscle pain)	21 (77,8)	6 (22,2)	0 (0,0)	0,233*
**Cognitive alterations**				
Perception	17 (85,0)	3 (15,0)	0 (0,0)	0,137*
Attention	52 (82,5)	10 (15,9)	1 (1,6)	0,006*
Memory	83 (75,5)	25 (22,7)	2 (1,8)	0,012*
	**Median**	**Median**	**Median**	
	**(P25; P75)**	**(P25; P75)**	**(P25; P75)**
	37	39	38	
Age	(33,0; 42,0)	(35,0; 45,0)	(33,0; 45,5)	**0,010^A^**
	10	10,5	11	
Time in PMERJ	(3,0; 16,0)	(3,0; 20,0)	(2,0; 22,0)	0,175^A^
	6	6	7,5	
Hours of sleep per day	(6,0; 7,0)	(6,0; 8,0)	(6,4; 8,0)	**0,001^A^**

*Chi-square.

**Fischer’s exact test. ^A^Kruskal-Wallis.

**There were participants who did not respond to some variables.

## DISCUSSION

Concurring with the results of this study, studies have shown that, in relation to sociodemographic aspects, there was greater participation by men^(13)^, with a prevalent age range of 30 to 39 years (42.1%)^(14)^, married or in a stable union, with a decreasing number of children, ranging from 0 to 4 children, with the majority (44.2%) having no children^(15)^, and greater adherence to the privates^(16)^.

Regarding cognitive abilities, they are more likely to decline in all domains as time passes^(17)^, including memory, perception, language, executive functions and attention^(18)^. Furthermore, cognitive function is the ability that suffers most with aging^(19)^, factors that are in line with the results of this study, where cognitive cost was significantly related to the age of the police officers.

Derived from this, sleep is another important aspect observed in the sociodemographic characteristics of health that merits discussion, since it is one of the basic needs of human beings and is directly related to quality of life^(20)^. According to the results of this study, hours of sleep were a factor that exacted a high cognitive cost among police officers, mainly due to the irregular duty roster, which is sometimes daytime and sometimes nighttime.

In line with this logic, a study carried out to assess the influence of sleep quality on the quality of life at work of military police officers from three municipalities in Bahia, concluded that the quality of life in the work environment was impaired by problems related to sleep and obtained the worst perception of quality of life in all dimensions: biological/physiological, psychological/behavioral, sociological/relational, economic/political, environmental/organizational^(19)^.

In regard to the classification of cognitive cost, of the ten items in the factor, eight were classified as severe and two as critical. A study that sought to understand the working conditions, processes and risk of illness in the female police battalion in Rio Grande do Norte, Brazil, also rated the cognitive factor as moderate to critical (μ = 3.52; SD = 1.39), particularly the sub-items “being forced to deal with unforeseen events” (μ = 4.2) and “using memory” (μ = 4.2)^(21)^.

On the other hand, in regard to the association between cognitive cost and sociodemographic variables, there was a close relationship between the location of the “most violent” battalions and cognitive demand.

In line with this finding, a study carried out with primary care health workers showed that workers’ mental health is directly affected by the conditions of the place where they work. Among the various problems, depression, anxiety and burnout syndrome were the most common^(22)^.

A comparative study of the levels and sources of organizational stress in police officers working in a police incident reporting center in the UK found that the level of stress was directly associated with physical and mental health, as well as substance abuse. As stress increased in all profiles, there was also a worsening in physical and mental health and an increase in substance abuse. In addition, sociodemographic factors and family interference at work predicted typologies of high organizational stress^(23)^.

The results found here were also observed in a study that sought to evaluate the human costs related to medical service in an emergency care unit. The research showed that the continuous cognitive demand at work is a major influence on the health-disease process, making it difficult for workers to manage strategies to overcome problems^(8)^.

In this sense, the high rates of police officers with serious levels of occupational stress at work as a result of the hostile environment lead to physical and mental vulnerability in the individual, group and organizational spheres^(4)^. In this way, it can be inferred that these are factors present in everyday police work, which are predictors of risks to workers’ health.

Taking into account the demands of police work, acute psychological stress significantly affects episodic memory, which consists of memory for events linked to a specific context. However, a study carried out with the aim of assessing pre- and post-stress self-reported levels of anxiety, with ninety-two students from Tufts University in Massachusetts, USA, revealed that stress improved semantic memory retrieval^(24)^.

The level of stress experienced by police officers on a daily basis is attributed to that which is a product of the environment (social pressures and coercions), generating significant psychological and physical effects, resulting in an imbalance in executive functions, memory, attention, psychomotricity and general cognitive impairment^(25)^.

With regard to obesity, it has taken a cognitive toll on police officers, where factors can trigger the disorder, including physical, emotional and cognitive factors. However, in addition to traditional factors, there are results related to cognitive, emotional and neurophysiological changes, such as anxiety and difficulty controlling appetite, which are important in this regard^(26)^. Anxiety is directly related to difficulty controlling binge eating and sleep quality in young adults aged between 20 and 44^(27)^, as in the results of this study.

Situations such as those described above can cause workers to become exhausted with consequences for their health, such as “anxiety, depression, panic disorder, reaching the final stage of exhaustion which is Burnout Syndrome”, known as physical and mental degradation^(28)^, which coincides with the situation found among the police officers under study. Bearing in mind that the reasons for leave reported by police officers were anxiety attacks (18%), depression (14.3%), panic disorder (4.7%), 3.8% bipolar disorder, 3.8% other mental disorders, 2.0% with a confirmed diagnosis of burnout syndrome and 1.3% drug addiction.

Although the percentages were low, they are significant, as they are capable of making this class of worker seriously ill. In view of this, the symptoms of these occupational illnesses also include cognitive problems, such as difficulty memorizing and concentrating, as well as sleep disorders, physical tiredness and aching^(29,30)^, in line with the results of this study.

In short, the police work process involves complex adversities, such as violence, organizational and social pressure, which demand a high cognitive cost. These factors result in pathogenic suffering that negatively impacts memory and concentration, as well as sleep disorders and neuropsychological conditions that trigger obesity and disproportionate reactions to work events in the day-to-day organization of work and the work process, which can lead to risks for all elements of the work process.

Seen from this perspective, and given the increasingly precarious nature of work^(2)^, it is necessary to think that certain changes and measures taken in relation to organizational management could contribute to improving the quality of life of military police workers and reduce the risks and illnesses arising from this process^(17)^.

The type of study can be considered a limitation, since a cross-sectional study restricts inferences about the influence between the variables investigated and does not allow for cause-and-effect statements between them. Longitudinal studies are therefore suggested to better understand the phenomenon at different points in time.

The results of the study have contributed to knowledge about the human cost of the work of military police officers in Rio de Janeiro, highlighting factors that contribute to the illness of the military police officers who took part in the study. These results can be presented to Military Police managers with a view to reviewing work contexts in order to improve service management. This could result in a reduction in human costs, meeting the health care and work demands of this group of workers, and in this way, improving the service provided to users of the Rio de Janeiro State Public Security Department.

## CONCLUSION

The results of the study led us to conclude that there was a serious risk of illness in terms of cognitive cost, where all the items indicated that the work context strongly contributes to the illness of police officers. There were also significant associations with obesity, cognitive alterations in attention and memory, age and hours of sleep.

It should be noted that the physical and affective costs were not classified as severe, but were classified as critical and worrying. Therefore, perhaps one of the greatest challenges is to rethink the configuration of the Military Police’s work organization in order to promote resilient and more flexible management, in the construction of identification, reception and intervention programs in the health sector.
